# Severe angioedema following dupilumab therapy for bullous pemphigoid

**DOI:** 10.1016/j.jdcr.2026.05.031

**Published:** 2026-05-29

**Authors:** Harisankeerth Mummareddy, Shannon Han, Whitney Shroyer

**Affiliations:** a Department of Dermatology, The University of Tennessee Health Science Center College of Medicine, Memphis, Tennessee; b Kaplan-Amonette Department of Dermatology, University of Tennessee Health Science Center, Memphis, Tennessee

**Keywords:** angioedema, bullous pemphigoid, dupilumab

## Introduction

Bullous pemphigoid (BP) is the most common autoimmune subepidermal blistering disorder and primarily affects older adults. It is characterized by autoantibodies against hemidesmosomal proteins that activate complement, induce inflammation, and lead to blister formation. Standard management includes high-potency topical corticosteroids and systemic immunosuppressive therapies, including oral corticosteroids, doxycycline, and steroid-sparing agents.^1^ However, many patients experience refractory disease, steroid dependence, or treatment-limiting adverse effects.

Dupilumab, a monoclonal antibody targeting the interleukin (IL)-4 receptor alpha subunit, inhibits IL-4 and IL-13 signaling and modulates type 2 inflammation. It was recently approved for moderate-to-severe BP and has demonstrated efficacy in reducing disease activity and steroid requirements.^2^^,^^3^ Clinical trials and real-world studies have generally supported a favorable safety profile, with injection-site reactions and ocular symptoms being most commonly reported.^4^

Although dupilumab is well tolerated in most patients, systemic hypersensitivity reactions, including angioedema, have been reported in post-marketing surveillance, but reports in BP remain limited.^5^ We describe a case of severe angioedema with oropharyngeal involvement shortly after initiation of dupilumab for refractory BP.

## Case report

A 77-year-old man with a several-year history of biopsy- and serology-confirmed BP initially presented with localized intermittent blistering that was responsive to high-potency topical corticosteroids. Over time, he developed generalized pruritic tense bullae and erosions involving the head, neck, trunk, and extremities without mucosal involvement. Despite maximized topical therapy, he experienced recurrent flares requiring multiple courses of systemic corticosteroids. Given persistent disease activity and steroid dependence, dupilumab was pursued for systemic control. He received a standard subcutaneous loading dose in the clinic, with plans for maintenance dosing every 2 weeks.

**Fig 1:**
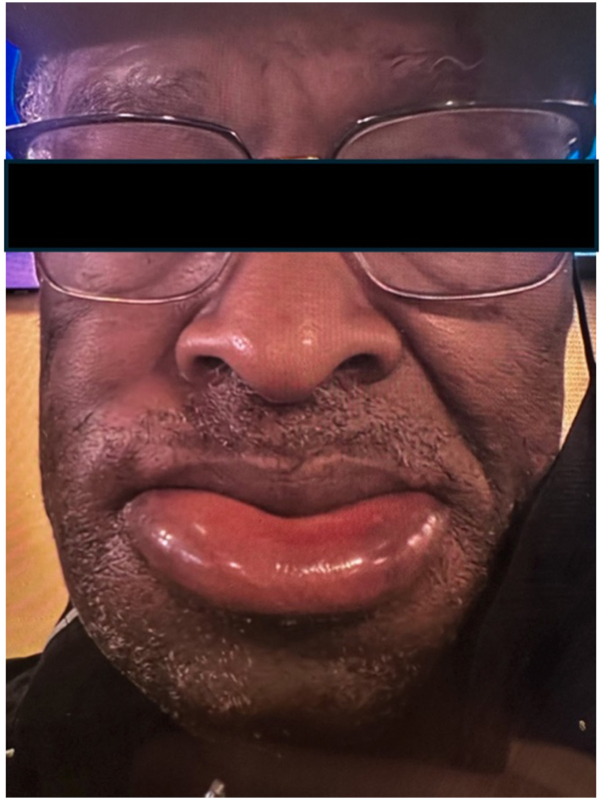
Facial swelling 3 days following the patient’s first Dupilumab injection.

Three days after receiving his first dupilumab injection, he developed abrupt swelling of the lower lip and chin (Fig 1), accompanied by hoarseness and subjective throat tightness. He experienced no rash or respiratory distress. His medical history was notable for hypertension, hyperlipidemia, prostate cancer in remission, and chronic leukopenia documented since 2016. Long-standing medications included losartan, olmesartan, atenolol, and atorvastatin, with no recent medication changes or prior known drug allergies. He denied use of nonsteroidal anti-inflammatory drugs. He denied any new foods or recent infections.

Physical examination in the emergency department was notable for swelling of the lip, tongue, and posterior oropharynx with no signs or symptoms of respiratory compromise. He was treated with intravenous corticosteroids, H1 and H2 antihistamines, and intramuscular epinephrine. Due to concern for progressive angioedema, fresh frozen plasma was administered, and he was admitted to an intensive care setting for close airway monitoring. Laboratory evaluation demonstrated mild leukopenia and anemia without eosinophilia. Serum creatinine was mildly elevated (1.24 mg/dL) and albumin was low (3.2 g/dL), both at chronic baseline; hepatic function was unremarkable. No complement levels (C3, C4, C1 esterase inhibitor) or mast cell mediator studies were obtained. His swelling improved over 24 to 48 hours with supportive care and corticosteroids.

Since discontinuing Dupilumab, the patient has not reported any recurrent episodes of facial swelling. He was eventually transitioned from systemic corticosteroid taper to a steroid-sparing immunosuppressant and referred to allergy and immunology for further workup.

## Discussion

This case describes severe angioedema with airway-adjacent involvement occurring shortly after the first exposure to dupilumab in a patient with refractory BP. Using the Naranjo Adverse Drug Reaction Probability Scale, we calculated a score of 4 (“possible” ADR); the WHO-UMC system similarly classified this as “possible” given the temporal relationship and positive dechallenge but competing causes.^6^ The temporal relationship between drug initiation and symptom onset, absence of prior similar episodes, and clinical improvement following discontinuation support a drug-associated reaction.

Angioedema is listed as a post-marketing immune system adverse reaction in the US prescribing information for dupilumab, though its true incidence remains unknown.^5^ Controlled clinical trials across multiple indications, including BP, did not identify angioedema as a prominent adverse event, suggesting that this reaction is rare.^4^

The differential diagnosis includes mast cell-mediated hypersensitivity, bradykinin-mediated angioedema, acquired C1 esterase inhibitor deficiency related to malignancy, and idiopathic angioedema. Long-standing use of 2 angiotensin receptor blockers (losartan and olmesartan) is a recognized confounder, as these agents are associated with angioedema, with an estimated incidence of 0.11%^7^ and African American patients experiencing approximately 1.5- to 2-fold higher risk for ARB-associated angioedema specifically.^8^ However, the absence of prior episodes despite over 15 years of antihypertensive use and the close temporal association with dupilumab initiation favor a drug-related mechanism. Atenolol and atorvastatin, also rarely associated with angioedema, had been used chronically without incident. The absence of complement testing precludes evaluation of complement-mediated mechanisms.

The mechanism underlying dupilumab-associated angioedema remains unclear. Transient eosinophilia, altered cytokine signaling, and immune dysregulation have been reported previously.^9^^,^^10^ In our patient, eosinophil counts were not elevated during the acute episode, and comprehensive immunologic testing was not performed, limiting mechanistic conclusions.

Only 2 prior cases of dupilumab-associated angioedema have been reported—one in a child^9^ and 1 in an adult.^10^ To our knowledge, this is the second reported case in an adult.

Our patient’s presentation is notable for occurring after the first injection, involvement of the posterior oropharynx with voice change, and the need for intensive monitoring. Additionally, this reaction occurred in the setting of BP, a recently approved indication for dupilumab, expanding the clinical contexts in which this adverse event may be encountered.

The severity of presentation and documented post-marketing signal warrant clinical awareness. Clinicians prescribing dupilumab for BP should counsel patients regarding symptoms of angioedema and advise prompt evaluation for facial or oropharyngeal swelling. Complement levels and tryptase should be obtained in the acute setting to aid mechanistic characterization. Dupilumab should be considered in the differential diagnosis of acute angioedema following recent injection, particularly in older adults with multiple comorbidities.

## Conflicts of interest

None disclosed.
